# Yokukansankachimpihange Is Useful to Treat Behavioral/Psychological Symptoms of Dementia

**DOI:** 10.3389/fnut.2020.529390

**Published:** 2021-01-21

**Authors:** Eiichi Katsumoto, Toru Ishida, Kenji Kinoshita, Miho Shimizu, Toshihito Tsutsumi, Yoko Nagai, Masakazu Nishimura, Toshio Yokouchi, Yasushi Yoshida

**Affiliations:** ^1^Osaka Association of Psychiatric Clinics, Osaka, Japan; ^2^Katsumoto Mental Clinic, Osaka, Japan; ^3^Ishida Clinic, Osaka, Japan; ^4^Kinoshita Clinic, Osaka, Japan; ^5^Shimizu Clinic, Osaka, Japan; ^6^Tsutsumi Clinic, Osaka, Japan; ^7^Nagai Clinic, Osaka, Japan; ^8^Nishimura Clinic, Osaka, Japan; ^9^Yokouchi Clinic, Osaka, Japan; ^10^Yoshida Clinic, Osaka, Japan

**Keywords:** multi-center research, behavioral dementia symptoms, psychological dementia symptoms, dementia, insomnia, Alzheimer's disease

## Abstract

Yokukansankachimpihange is a Japanese herbal medicine reported to benefit anxiety and sleep disorders, and it has recently been introduced to treat behavioral and psychological symptoms of dementia. There are no multicenter studies of its effectiveness regarding dementia in Japan, and this study's main objective was to clarify the effects of Yokukansankachimpihange on behavioral and psychological symptoms of dementia in a sample of patients from multiple healthcare centers. Nine facilities affiliated with Osaka Association of Psychiatric Clinics participated in November 2013 through April 2015 and provided 32 Alzheimer's disease patients to whom Yokukansankachimpihange was orally administered for 8 weeks. During the study, the patients continued their regular medication regimens. Behavioral and psychological symptoms of dementia (Behavioral Pathology in Alzheimer's Disease Rating Scale [Behave-AD]), core symptoms [Mini-Mental State Examination (MMSE)], activities of daily living [Nishimura Activity of Daily Living Scale (N-ADL)], and gastrointestinal symptoms (nausea/vomiting, loss of appetite, gastric discomfort, constipation, and diarrhea) were measured at baseline, after 4 weeks of treatment and after 8 weeks of treatment. Yokukansankachimpihange was orally administered at a dosage of 7.5 g twice daily before or between meals for 8 weeks. The Behave-AD mean score significantly improved after 8 weeks of treatment. There were no significant changes in MMSE, N-ADL, or gastrointestinal symptoms; however, decreased gastrointestinal scores were observed after 8 weeks. There were no side effects related to Yokukansankachinpihange. Pharmaceutical treatments are important for treating behavioral and psychological symptoms of dementia, and this study confirmed Yokukansankachimpihange's efficacy for treating Alzheimer's disease. Because the aggressiveness and sleep disorder components of the Behave-AD construct were the symptoms most improved and those symptoms are known to significantly burden dementia patients' caregivers, Yokukansankachimpihange's efficacy might indirectly relieve these caregivers' burden of care.

## Introduction

Antipsychotics used to treat behavioral and psychological symptoms of dementia (BPSD) require particularly careful administration because of their potential side effects. Their main side effects are extrapyramidal symptoms, oversedation, cognitive decline, increased cerebrovascular disorders, and increased risk of mortality. A risk of increased blood sugar levels needs to be considered in the use of atypical antipsychotics. Avoiding typical antipsychotics as much as possible and limited treatment with atypical antipsychotics have been recommended (1).

Recently, Yokukansan (YKS) (2), a Japanese herbal medicine, has been used to treat BPSD. Yokukansankachimpihange (YKSCH) is a Japanese herbal medicine made up of *Citrus unshiu* peel and *Pinellia tuber* combined with YKS. *Citrus unshiu* peel and *Pinellia tuber* were formulated to improve gastrointestinal symptoms (GIS), such as loss of appetite, nausea, and vomiting (3). The blend is expected to favorably influence anxiety and sleep disorders because of the influence that *Citrus unshiu* peel has on serotoninergic neural pathways (4).

Several previous clinical studies have examined the effects of YKSCH on BPSD (5–9); however, most of them were single-setting studies and none involved multiple healthcare centers. Further, the only cholinesterase inhibitor (ChEI), which is the only class of medications on the market currently being used to treat dementia, evaluated by the previous studies was donepezil. This study aimed to clarify the effects of YKSCH on BPSD in a nine-center joint clinical study under conditions similar to those in which Alzheimer's disease (AD) medications are used in real-world settings without specifying the type and route of ChEI administration.

## Methods and Materials

### Setting and Participants

This multi-center prospective clinical study initially recruited 53 patients diagnosed with AD who met the inclusion and exclusion criteria. It was conducted between November 2013 and April 2015 at nine facilities affiliated with the Osaka Association of Psychiatric Clinics, Osaka, Japan.

The inclusion criteria were AD patients (1) currently on a ChEI that lacked efficacy for their BPSD, (2) who had difficulty continuing the ChEI treatment because of gastrointestinal side effects, (3) who had no ChEI currently administered with a demonstrated need for BPSD treatment, or (4) on continuous ChEI treatment for more than 4 weeks without a change in medication type or dosage.

The exclusion criteria were (1) patients currently on memantine or YKS or (2) patients who had recently started or changed the dosage of any medication that might influence the efficacy of YKSCH, such as ChEI, antipsychotics, or Japanese herbal medicines, within 4 weeks before the study began.

### Ethics

The Ethics Committee of the Osaka Association of Psychiatric Clinics approved this study. Written informed consent was obtained from all the enrolled patients.

### Procedure

YKSCH was orally administered at a dosage of 7.5 g twice daily before or between meals for 8 weeks. All other medications were continuously administered without changing the dosages, and no additional medications were administered during the study period. Four outcome variables were assessed at baseline, 4 weeks into the YKSCH treatment, and 8 weeks into the YKSCH treatment.

### Variables

BPSD was measured by responses to the Behavioral Pathology in Alzheimer's Disease Rating Scale (Behave-AD) using the total Behave-AD score, which was based on responses to 25 items, with a maximum score of 75 points. The item measuring “care burden” was excluded from the calculation of Behave-AD score, because it was an overall indicator of BPSD. The 11-item Mini-Mental State Examination (MMSE) was used to measure core symptoms on a 30-point scale. Activities of daily living were evaluated using the N-type older adult living activities scale (Nishimura Activity of Daily Living Scale; N-ADL), a five-item 50-point scale. Four levels of GIS regarding nausea and vomiting, loss of appetite, stomach discomfort, constipation, and diarrhea were identified where 0 = no symptoms, 1 = some untroublesome symptoms, 2 = some symptoms that do not interfere with activities of daily living, and 3 = symptoms that interfere with activities of daily living. The patients assessed the severity of their symptoms on a questionnaire survey using a 15-point scale. Higher scores on the Behave-AD and GIS and lower scores on the MMSE and N-ADL indicated more severe symptoms/disabilities. The four variables (Behave-AD, MMSE, N-ADL, and GIS) were measured before YKSCH administration (baseline), 4 weeks into the YKSCH treatment, and 8 weeks into the YKSCH treatment.

### Statistical Analysis

Multiple comparison tests were performed using one-way analysis of variance (ANOVA) and Tukey's HSD test, with a statistical significance cutoff level of *p* < 0.05, to assess the influences of YKSCH on the four outcomes after 4 weeks and after 8 weeks.

## Results

At baseline, 53 cases at nine institutions were enrolled in the study. Two patients did not visit the hospital after the baseline, 12 patients violated the protocol, and the safety analysis of the influence of YKSCH was assessed for the remaining 39 patients. The 12 protocol violations (some cases had more than one violation) were two cases of memantine use, six cases of concomitant use of a ChEI initiated at the start of the study, six cases of concomitant use of non-ChEI medications started within 1 month of the study, and three cases of additional use of concomitant medications during the study period. The efficacy analysis was performed on 32 patients because of the loss of two patients who refused to take the medication, four patients who did not participate in the 8-week follow-up, and one patient who independently discontinued treatment due to perceived ineffectiveness. Of the 32 patients, nine were male and 23 were female. The average age was 76.9 ± 9.4 years. Table 1 describes the personal characteristics of the 32 patients in the efficacy analysis. No side effects deemed attributable to the treatment were observed.

**Table 1:** Sample characteristics.

**Characteristic**	**Mean ± SD**
Age (years)	76.9 ± 9.4
Height (cm)	156.7 ± 6.5
Weight (kg) (*n* = 13)^a^	53.1 ± 10
Dementia history (months)	49.4 ± 40.5
**Gender**	**Number of cases (%)**
Male	9 (28)
Female	23 (72)
Total	32 (100)
**Medical history**	**Number of cases (%)**
None	21 (66)
Existing	11 (34)
Total	32 (100)
Disease^b^	
Hypertension	1
Hyperlipidemia	1
Glaucoma	1
Cholecystitis	1
Alcoholism	1
Cerebral infarction	1
Epidural hematoma	1
Lumbar compression fracture	1
Previous spinal canal stenosis surgery	1
Breast cancer	1
Previous breast cancer surgery	1
Previous stomach cancer surgery	1
**Medical complications**	**Number of cases (%)**
None	16 (50)
Existing	16 (50)
Total	32 (100)
Complication^b^	
Hypertension	8
Diabetes	3
Hyperlipidemia	2
Prostatic hypertrophy	2
Depressive state	2
Hyperuricemia	1
Chronic gastritis	1
Chronic nephritis	1
Primary osteoporosis	1
Hypothyroidism	1
Tension headache	1
Depression	1
Suspected Parkinsonism	1
**Concomitant AD medication**	**Number of cases (%)**
Yes	25 (78)
No	7 (22)
Total	32 (100)
**Concomitant ChEI medication**	**Number of cases (%)**
None	12 (38)
Existing^c^	20 (62)
Total	32 (100)
Medication^b^	
Donepezil (5 mg)	9
Donepezil (10 mg)	2
Galantamine (8 mg)	1
Galantamine (16 mg)	3
Galantamine (24 mg)	1
Rivastigmine (18 mg)	4
**Other concomitant medications for BPSD**	**Number of cases (%)**
None	22 (69)
Existing	10 (31)
Total	32 (100)
Medication^b^	
Antipsychotic	7
Antidepressant	5
Antianxiety	4
Hypnotic	1

a Missing data = 19.

b There are duplicate cases.

c *Administration period until start of study, 9.9 ± 9.7 months [n = 16]*.

### YKSCH Results

**Figure 1:**
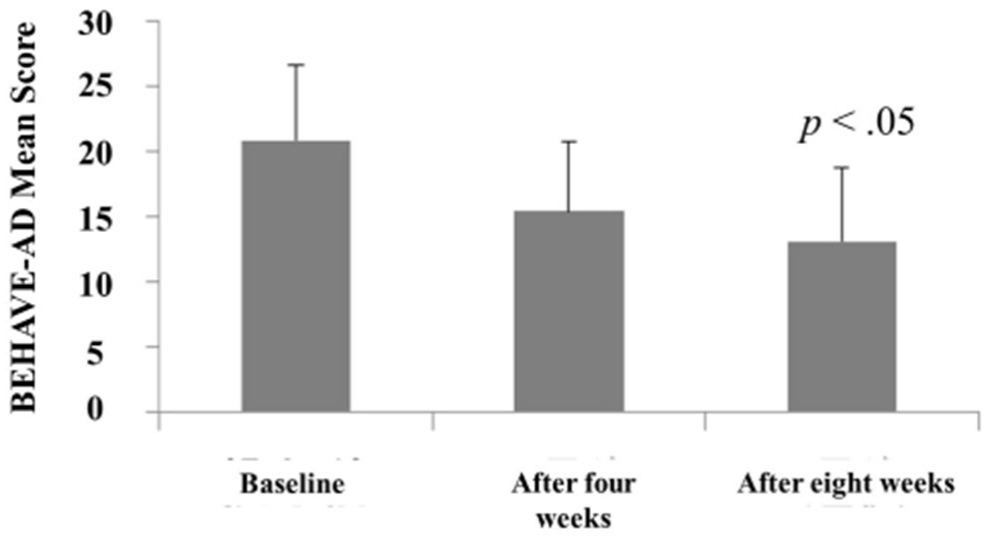
The effects of YKSCH on BPSD (*n* = 29). Data were Behave-AD scores (means ± SD and ranges) after YKSCH administration. Three cases with missing data were dropped from the analysis. *Post hoc* Tukey's HSD tests were compared to baseline.

**Figure 2:**
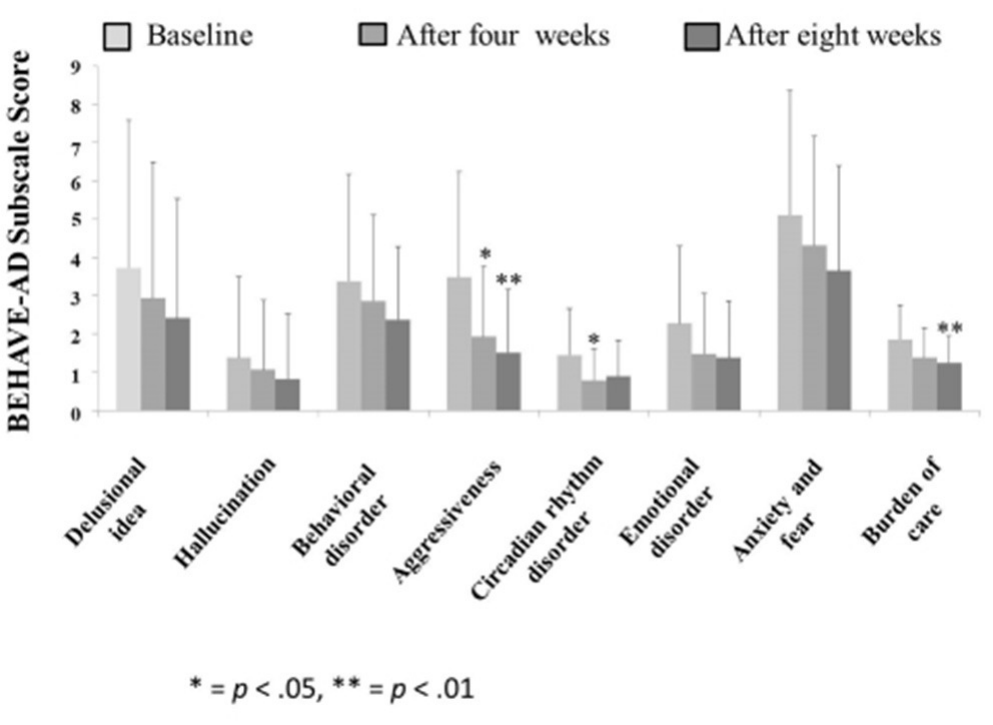
The effects of YKSCH on BPSD items. Data were Behave-AD item scores (means ± SD and ranges) after YKSCH administration. Results were on 29 cases regarding “burden of care” and 28 cases otherwise. All excluded cases were dropped because of missing data. *Ad hoc* multiple comparison Tukey's HSD tests (*p < 0.05, **p < 0.01) were compared to baseline.

**Figure 3:**
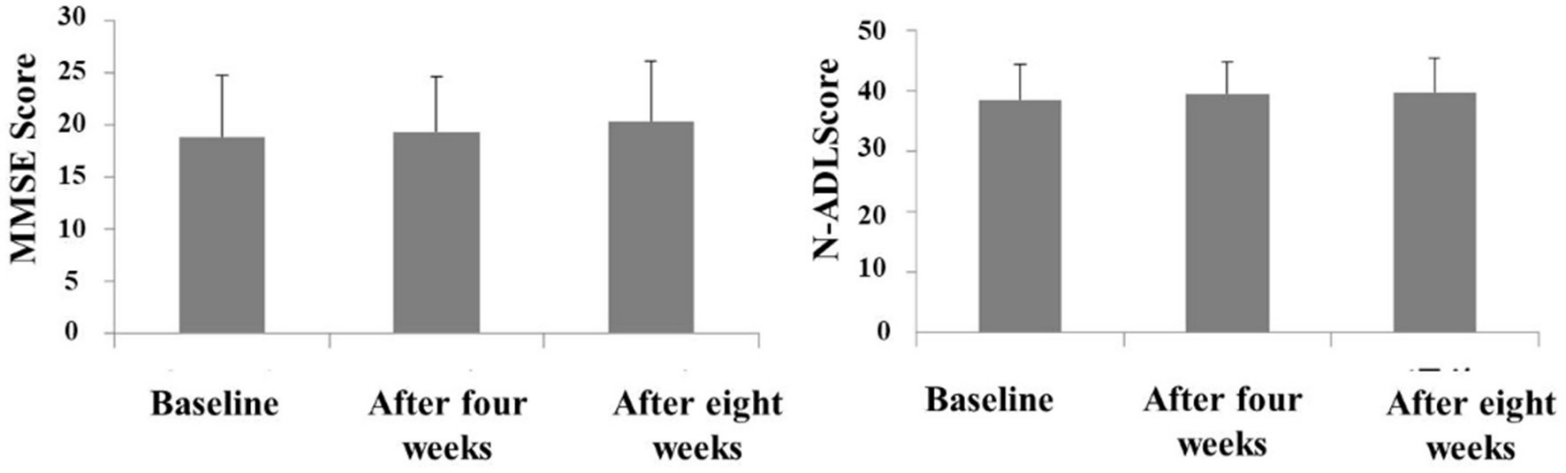
The effects of YKSCH on MMSE and N-ADL. Data were MMSE scores and N-ADL scores (means ± SD and ranges) after YKSCH administration. MMSE results were on 20 cases (12 cases dropped because of missing data), and N-ADL results were on 21 cases (11 cases dropped because of missing data).

**Figure 4:**
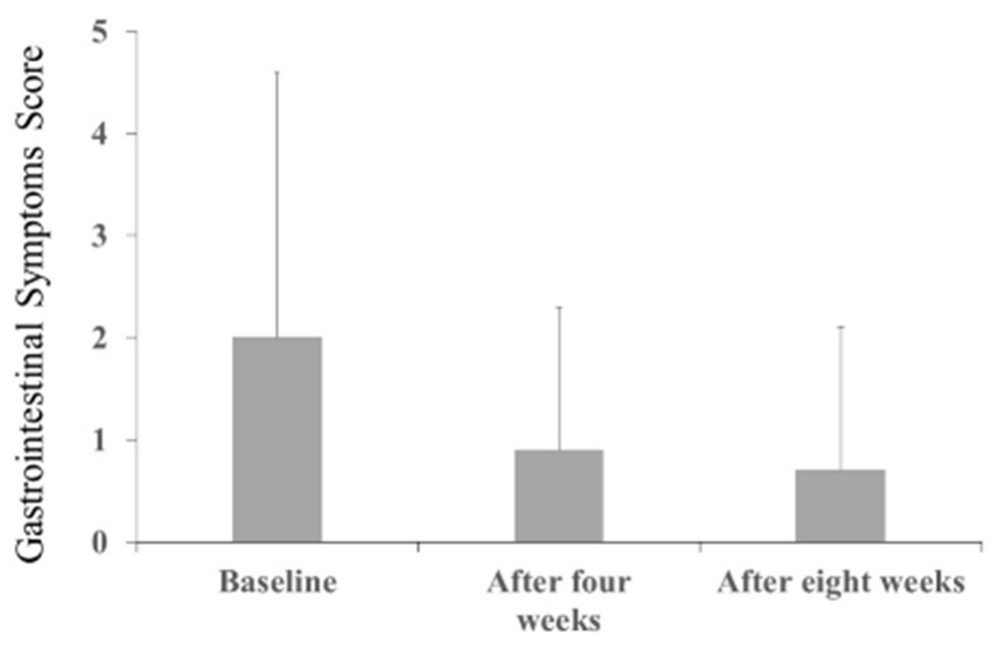
The effects of YKSCH on Gastrointestinal Symptoms (*n* = 28). Data were gastrointestinal symptoms scores (means ± SD and ranges) after YKSCH administration. Four cases with missing data were dropped from the analysis. *Post hoc* Tukey's HSD tests were compared to baseline.

The Behave-AD mean score was 20.8 ± 11.4 at baseline, 15.4 ± 10.4 4 weeks later, and 13.1 ± 9.1 after 8 treatment weeks. An improvement was observed between baseline and 8 weeks (Figure 1). Of the construct's 25 items, “aggression” significantly improved after 4 and after 8 weeks of treatment, and “circadian rhythm disorder” was significantly improved after 4 weeks of treatment. The “care burden” indicator also was significantly reduced after 8 weeks of treatment (Figure 2). Core symptoms (MMSE) and activities of daily living (N-ADL) did not significantly change during the treatment period (Figure 3). Regarding GIS, 15 patients reported symptoms at baseline [mean 2.0 ± 2.6 (range: 1–8)]. The score decreased to 0.9 ± 1.4 (range: 1–9) after 4 weeks of treatment and to 0.7 ± 1.4 after 8 weeks of treatment, but there was no significant difference from baseline (Figure 4). Seven of 15 patients had symptoms at the start of the study that disappeared after 8 weeks of treatment.

## Discussion

YKSCH was administered for 8 weeks to 32 AD patients who were not experiencing sufficient efficacy with conventional BPSD treatments or had difficulties with continuous use of a ChEI due to gastrointestinal side effects. Because improvement was observed in 8 weeks in the previous study (6), the administration period was set to 8 weeks in this study as well. The results indicated significant improvement in two Behave-AD items (aggression and circadian rhythm disorders) and in the Behave-AD overall care burden score. MMSE and N-ADL mean scores were not significantly influenced.

YKSCH is a blend of *Citrus unshiu* peel and *Pinellia tuber* with YKS. YKS is a prescription medication developed to cure oversensitivity conditions, including children crying at night. *Citrus unshiu* peel and *Pinellia tuber* are used to improve GIS, such as loss of appetite and nausea/vomiting (3); thus, it is suitable for older adults with reduced digestive function. In this study's analysis of GIS, the mean score improved, but the differences were not statistically significant. YKSCH might have contributed to improving GIS because *Citrus unshiu* peel and *Pinellia tuber* have been reported to improve loss of appetite (10) caused by suppression of cisplatin-induced anorexia via 5-HT_2_ receptor antagonism.

The “aggression” item in Behave-AD aims to assess the extent of rants, threats, violence, and restlessness. This BPSD symptom was the most improved in response to YKSCH treatment. Basic research has investigated the mechanism by which YKS diminishes aggression (11). It has been hypothesized that YKS affects glutamatergic neural pathways related to aggressiveness and serotoninergic (5-HT) pathways related to impulsivity (11). It also has been reported that Uncaria hook, a component of YKSCH, has a 5-HT_1A_ receptor-stimulating action and a 5-HT_2A_ receptor-blocking action as well as an ability to normalize the extracellular fluid concentration of glutamate (11), all of which might be expected to contribute to reductions in aggression.

The “circadian rhythm disorder” Behave-AD item is focused on sleep-wake disorders (insomnia). High rates of insomnia are observed in patients with dementia, which not only significantly affects patients' quality of life, but also increases the burden of care because it causes physical and mental stress for the caregiver. Regarding YKSCH's mechanism of action on insomnia, basic research has suggested that Uncaria hook (11) and *Citrus unshiu* peel (4) have effects on nervous system serotonin and that YKS influences the GABA_A_-benzodiazepine receptor complex (12). We found that YKSCH treatment alleviated the severity of circadian rhythm disorder, i.e., sleep-wake disorder, implying that YKSCH might be effective for nighttime insomnia and improving quality of life, which, in turn, suggests that the mental and physical burden on caregivers might be reduced by suppressing patients' nighttime activity levels.

Because aggressiveness and circadian rhythm disorder are major contributors to caregivers' burden, alleviating both problems likely led to a reduction in the burden of care. The results suggest that YKSCH might be an effective treatment for patients who demonstrate significant aggressiveness and those who struggle with insomnia. There were no side effects of the administration of this medication observed throughout the study. Although this study has some limitations, such as the lack of a control group and the insufficient number of cases, YKSCH is considered a suitable treatment for AD sufferers.

## Conclusions

This study clarifies the efficacy of YKSCH for BPSD. The results suggest that treatment with YKSCH, a Japanese herbal medicine, might benefit AD patients and their caregivers.

## Data Availability Statement

The raw data supporting the conclusions of this article will be made available by the authors, without undue reservation, to any qualified researcher on request.

## Ethics Statement

The studies involving human participants were reviewed and approved by The Osaka Association of Psychiatric Clinics. The patients/participants provided their written informed consent to participate in this study.

## Author Contributions

TI designed the study plan and EK, TI, KK, MS, TT, YN, MN, TY, and YY registered the cases. EK conducted the analysis and prepared the manuscript. All authors contributed to the article and approved the submitted version.

## Conflict of Interest

The authors declare that the research was conducted in the absence of any commercial or financial relationships that could be construed as a potential conflict of interest.
